# Supplementary material for: The development of new occupation practitioners in China’s first-tier cities: A comparative analysis
Source: PLoS One. 2024 Jan 25;19(1):e0284148. doi: 10.1371/journal.pone.0284148 (PMC10810496; doi:10.1371/journal.pone.0284148)
Supplement: Attachment — Submitted filename: Response to Reviewers.docx [file pone.0284148.s003.docx]

**Dear editors and reviewers,**

We would like to thank you and the two reviewers and editors for their valuable comments and kind suggestions regarding the manuscript we submitted. These comments and suggestions directed us to improve the content of our study. We have revised our paper again, and the revised version of the manuscript incorporates the comments and suggestions of the reviewers.

The details of modification were listed point by point:

**Response to Reviewer #1:**

**[Comment 1]** The current literature review is mostly from Chinese authors, which is very lacking in international perspective. Please revisit the more diverse international literature and strengthen the critical literature review.

**[Response]** *Thank you very much for your comments, which is highly appreciated.* *In the new occupation section of the literature review, we have also added relevant research by authors from other countries and, at the same time, added critical perspectives.*

*“Initially, the United States was the country with the best new career development because its culture emphasized independence and self-realization, and its labor market practices were quite flexible and open (Pringle and Mallon，2003；Mayhofer et al.，2004). But today's society is becoming more and more "mobile" and uncertain (Bauman, 2007). Other countries are also interested in this (Druker and Stanworth 2004).”*

*“Such a survey is important because it is an important source of job satisfaction (Dawis and Lofquist, 1984).”*

**[Comment 2]** After reporting your results, compare your results with similar studies. Maybe a discussion section could be added.

**[Response]** *Thank you for pointing out this problem in the manuscript. We have added a discussion section to compare it with the research of other scholars. First of all, this study is summarized. Then, for some research conclusions, we compared with the previous related research. This highlights the significance of our research. Thank you again for your valuable advice. The detailed discussion is as follows.*

*“To better evaluate the development status of new occupation practitioners in China, discover relevant issues during development, and provide a reference for the development of a digital economy, this study measured the development level of new occupation practitioners in 19 “first-tier” cities in China from five dimensions: group size, cultural literacy, salary level, occupation perception, and environmental perception. Based on a comparative analysis of 19 “first-tier” cities, this study describes the development level of new occupation practitioners in China. The results indicate that the new occupation practitioners have a high degree of recognition of and satisfaction with the new occupations and hold a positive and optimistic attitude toward the development prospects of the new occupation practitioners. The four first-tier cities lead 15 new first-tier cities in the development level of new occupation practitioners. Among the 15 new first-tier cities, Chengdu and Hangzhou did well in the development of new occupation practitioners. Comparatively, other new first-tier cities have a relatively slow start and late development of new occupations. In most cities, the development of new occupation practitioners is not coordinated with the five indicators, severely affecting the healthy development of new occupation practitioners. The policy strength of relevant departments in some cities is low and has not taken the shape of a good policy environment and social environment to promote the sustainable development of new occupation practitioners.*

*In this study, most new occupations are borderless. Their work is very flexible. For example, online delivery staff and ride-hailing drivers can work according to their own time. There are fewer restrictions on working hours and workplaces. They are more likely to have multiple jobs (Sprajcer, 2021), and at the same time, they tend to be younger (Sprajcer, 2021); this is consistent with our research results. New occupation practitioners are more satisfied with their careers, and Kundi (2020) showed that this kind of career practitioner has higher satisfaction.*

*The results show that new occupation practitioners in the four first-tier cities have a higher level of development. Cities with better economic development are more conducive to developing new occupations. The new occupation is the product of conforming to the development of the times, and cities with better economic development can provide a more favorable environment for the development of the new occupation. A long time ago, some scholars proposed that borderlessness would soon become the main career model (Arthur and Rousseau, 1996), which has become a reality. In recent years, its scale has expanded. An increasing number of people benefit from such careers (Handy, 1989).”*

**[Comment 3]** At present, the language of this manuscript is not standardized and fluent. Please make sure this manuscript has been checked by a native English-speaking professor.

**[Response]** *Thank you for your valuable advice. This article has been proofread by a professional body for language and grammar. The proof material is shown in Figure 1.*

**Response to Reviewer #2:**

**[Comment 1]** I feel that you have addressed the reviewer comments sufficiently.My only substantive comment is that the whole manuscript needs to be language and grammar proofed by a native English speaker before publication. Please check your headings and sub-headings for grammatical soundness - for example, "2.1 New economic" doesn't sound right - should it be "New economy"?

**[Response]** *Thank you for your valuable advice.* We have corrected the problem you mentioned above. *This article has been proofread by a professional body for language and grammar. The proof material is shown in Figure 1.*

Figure 1 proof material
