# Supplementary material for: The development of new occupation practitioners in China’s first-tier cities: A comparative analysis
Source: PLoS One. 2024 Jan 25;19(1):e0284148. doi: 10.1371/journal.pone.0284148 (PMC10810496; doi:10.1371/journal.pone.0284148)
Supplement: Attachment — Submitted filename: Response to Reviewers.docx [file pone.0284148.s004.docx]

**Dear editors and reviewers，**

We would like to thank you and the two reviewers and editors for their valuable comments and kind suggestions regarding the manuscript we submitted. These comments and suggestions directed us to improve the content of our study. We have revised our paper substantially, and the revised version of the manuscript incorporates the comments and suggestions of the reviewers.

The details of modification were listed point by point:

**Response to Reviewer #1:**

**Question 1**

*1. The manuscript lacks a critical review of the latest literature published in 2022.*

**Response:**

Thank you for your valuable advice. According to your suggestion, we have added some relevant latest literature published in 2022 in section 2. Part of the added literature is shown below.

[23] Wang, Y., Wei YD., & Sun, BD., 2022. New economy and national city size distribution. Habitat International, 127, 102632.
[24] Nham, NTH., & Ha L., 2022. Making the circular economy digital or the digital economy circular? Empirical evidence from the European region. Technology in Society, 70: 102023.
[25] Li, ZG., & Wang J., 2022. The Dynamic Impact of Digital Economy on Carbon Emission Reduction: Evidence City-level Empirical Data in China. Journal of Cleaner Production, 351: 131570.
[28] Lederman, D., & Zouaidi, M., 2022. Incidence of the Digital Economy and Frictional Unemployment: International Evidence. Applied Economics, 54(51): 5873-5888.
[45] Mousa, M., & Chaouali, W., 2022. Job crafting, meaningfulness and affective commitment by gig workers towards crowdsourcing platforms. Personnel Review. DOI: 10.1108/PR-07-2021-0495.

**Question 2**

*2. The language of this manuscript is not standard and fluent. There are some spelling* *mistakes that have not been corrected. Please check the upper and lower case of each section.*

**Response:**

Thank you for raising this question. According to your suggestion, We have carefully checked the full text for spelling and grammar mistakes. And we would like to thank Editage (www.editage.cn) for English language editing. Please see the attachment for the proof.

**Question 3**

*3. Conclusions and discussion should be split into 2 sections.*

**Response:**

Thank you for your valuable advice. According to your suggestion, the conclusion and discussion have been divided into two separate sections.

**Response to Reviewer #2:**

*The authors appear to have responded to the feedback.*

*Please make sure that you have a good language proof of the manuscript*

**Response:**

Thank you for your valuable advice. We have carefully checked the full text for spelling and grammar mistakes. And we would like to thank Editage (www.editage.cn) for English language editing. Please see the attachment for the proof.

Sincerely thanks again to the editor and reviewers for their valuable comments on this version of this paper.

**Attachment**
