# Supplementary material for: The development of new occupation practitioners in China’s first-tier cities: A comparative analysis
Source: PLoS One. 2024 Jan 25;19(1):e0284148. doi: 10.1371/journal.pone.0284148 (PMC10810496; doi:10.1371/journal.pone.0284148)
Supplement: Attachment — Submitted filename: Response to Reviewers.docx [file pone.0284148.s005.docx]

**Dear editors and reviewers,**

Thank you very much for your valuable comments and kind suggestions regarding our research paper. Each of your feedback to strengthen our manuscript and we corrected point by point the manuscript accordingly. Your comments are in bold text and our responses in plain italics. The revised version contains your comments and suggestions, as well as recorded the changed below.

**Response to Reviewer #1:**

**[Comment 1]** After reading the revised version, I am quite satisfied. The authors have carefully revised the manuscript and the present version is acceptable.

**[Response]** *First of all, thank you very much for your affirmation of our work. Thank you again for your valuable suggestions in the manuscript review, which has greatly improved the quality of our paper.*

**Response to Reviewer #3:**

**[Comment 1]** Your references are somehow old, and I can even find some in the last century. I suggest you update your references. For instance, in Line 49-51, you can refer to the following papers: Research Progress of Green Marketing in Sustainable Consumption based on CiteSpace Analysis; Line 128: Digital economy is of great significance to carbon emission reduction[X]; X: Bibliometric review of carbon neutrality with CiteSpace: evolution, trends, and framework; 47, 60, 61 reference should be changed; they are too old.

**[Response]** *Thanks for all your comments and suggestions. We have made your recommended changes. According to your proposed, we cited two papers you mentioned and updated the relatively old references.*

*“[4] Geng, YQ. & Maimaituerxun, M., 2022. Research Progress of Green Marketing in Sustainable Consumption based on CiteSpace Analysis. SAGE Open, 12(3), 1-19.”*

*“[26] Geng, YQ., Zhu, RJ. & Maimaituerxun, M., 2022. Bibliometric review of carbon neutrality with CiteSpace: evolution, trends, and framework. Environmental Science and Pollution Research, 29(51), 76668-76686.”*

*”* *[49] Yukl, G., Mahsud,R., Prussia, G. & Hassan, S., 2019. Effectiveness of broad and specific leadership behaviors. Personnel Review, 48(3), 774-783.”*

*“[62] Inkson, K., Gunz, H., Ganesh, S. & Roper, J., 2012. Boundaryless Careers: Bringing Back Boundaries. Organization Studies, 33(3), 323-340.”*

*“[63] Kundi, YM., Hollet-Haudebert, S. & Peterson, J., 2021. Linking Protean and Boundaryless Career Attitudes to Subjective Career Success: A Serial Mediation Model. Journal of Career Assessment, 29(2), 263-282.”*

**[Comment 2]** You had better look for a professional language editing service (such as AJE or other PLOS certified services) to enhance your language. There are so many Chinglish and redundant expressions.

**[Response]** *Thank you for your valuable advice. We have corrected the problem you mentioned above. This article has proofread by a professional body for language and grammar. We revised the introduction、finding、discussion and conclusion.*

**[Comment 3]** Please redraw figure 1: current version is not clear (I am not sure whether because of the system's compression, or it is actually not clear). Maybe you can add different colors in different dimensions to make it more beautiful.

**[Response]** *Thank you for your kind suggestions. As you mentioned, the old one is not clear, we added other colors to differentiate. As shown in Figure1.*

Figure 1 Index system of new occupation practitioners
